# Safety in numbers? Evidence of non-social behaviour in the moon jellyfish *Aurelia spp.*

**DOI:** 10.1007/s10071-025-02023-3

**Published:** 2025-12-09

**Authors:** Alessandra Pecunioso, Christian Agrillo

**Affiliations:** 1 Department of General Psychology, University of Padova, Via Venezia 8, 35131 Padova, Italy; 2 Padua Neuroscience Center, Padova, Italy

**Keywords:** Cnidarias, Social behavior, Shoal choice, Embodied cognition

## Abstract

When animals explore a novel environment, they often aggregate in groups rather than staying isolated to reduce the risks of predation. This effect is known as ‘safety in numbers’ and has been widely reported in terrestrial and aquatic species. Jellyfish are often found to be clustered in the same areas in seas and oceans. Here, we asked whether one reason for such behavior might be the tendency to actively join other conspecifics when encountering novel environments. We observed moon jellyfish (*Aurelia* spp.) in a free-choice test measuring the proportion of time spent near conspecifics when they are inserted into an unfamiliar environment. We found evidence that jellyfish detect the presence of other conspecifics and use this information to actively direct their behavior in a given direction. Contrary to what was expected by the ‘safety in numbers’ effect, moon jellyfish have significantly distanced themselves from their social companions throughout the whole observation period. This result suggests that, although moon jellyfish are commonly observed in large aggregates in nature due to various environmental factors, they may exhibit limited social behavior in unfamiliar environments.

**Supplementary Information:**

The online version contains supplementary material available at 10.1007/s10071-025-02023-3.

## Introduction

The study of social cognition traditionally focuses on how animals perceive, process, and respond to social information, including how they make social decisions based on both conspecifics’ cues and ecological demands (Shettleworth 2009). In relation to this, living in groups involves both advantages and disadvantages depending on the context. Group living can present significant challenges, such as increased competition for food and mates, and a higher risk of disease transmission. However, despite these costs, social aggregation also offers important benefits, including enhanced foraging success, increased opportunities for social learning, and improved reproductive prospects (Alcock 2009). A major benefit of social behavior is the increased protection it offers from predation. When exploring a novel environment, social animals often aggregate to increase individual vigilance and the chance to spot potential predators in the surroundings. This anti-predator strategy becomes more effective when more conspecifics are present. This effect is named ‘safety in numbers’ and is widespread among animals (e.g., Foster 2017; Lehtonen and Jaatinen 2016). To evaluate whether this effect occurs in a given species, ethologists employ tracking methods to analyze how animals distribute themselves in their natural habitat when navigating potentially hazardous environments (Kays et al. 2015). In laboratory settings, they observe animals in free-choice tests when placed in a novel environment with conspecifics on one side of the experimental arena. The other side may present either no conspecifics or a smaller number of conspecifics (Freeberg et al. 2024; Hager and Helfman 1991a, b).

The tendency to stick to social companions when exploring novel environments is also observed in the aquatic species (Agrillo et al. 2012; Queiroz and Magurran 2005; Wong and Rosenthal 2005). Most studies focused on fish, while observations on aquatic invertebrates are rarer (Ferreira and Moita 2020; Jeanson and Deneubourg 2007). Cnidarians represent one of the largest animal phyla, with jellyfish being among their most prominent representatives. In particular, the moon jellies are globally distributed scyphozoans in seas and oceans. Predators of this species include fish, turtles, crustaceans, sea anemones, and corals. As an anti-predator strategy, moon jellyfish use stinging cells located in their tentacles, which contain specialized structures called nematocysts that release venom upon activation. However, jellyfish, including *Aurelia* spp., have also been observed forming large aggregates, also named as blooms (Hamner and Dawson 2009; Dong et al. 2010; Churnside et al. 2016). The causes of jellyfish blooms are not fully understood. Several well-documented ecological factors may contribute to these aggregations, including simultaneous strobilation (i.e., many polyps releasing ephyrae at the same time, Purcell et al. 2009; Liu et al. 2008), high genetic relatedness within blooms (due to individuals originating from a common polyp population, Aglieri et al. 2014), and environmental drivers such as currents, temperature, light cues, and prey availability (Syazwan et al. 2025; Graham et al. 2001). Beyond these established ecological explanations, we cannot exclude the possibility that social dynamics—such as those observed in both vertebrates and invertebrates—may also influence jellyfish behavior. For example, when exploring novel environments, moon jellyfish may benefit from the ‘safety in numbers’ effect, potentially favoring an active tendency to join conspecifics. There is evidence that larvae of moon jellyfish preferentially settle in locations already inhabited by conspecifics by using chemical signals, possibly to prevent settlement by competing species (Gröndahl, 1989). However, no information is currently available for the medusa stage and it remains unclear whether the aggregations observed in the wild result from active social attraction. Assessing jellyfish’s distribution in their natural habitat is challenging due to their patchy spatial distribution, both horizontally and vertically. Moreover, their gelatinous bodies make tagging difficult (Purcell, 2009), and the high-water content in their tissues further complicates acoustic sampling. For these reasons, laboratory studies are essential to understand their active responses to social stimuli when exploring novel environments.

To determine whether jellyfish blooms may involve an active behavioral component—separate from environmental factors such as chemical cues, temperature, oxygen, and salinity that can trigger strobilation in many polyps—this study adopted a procedure commonly used to assess fish behavior during exploration of unfamiliar environments (Agrillo et al. 2012; Piffer et al. 2012; Wong and Rosenthal 2005; Gatto et al. 2019). Recent studies showed that jellyfish display cognitive abilities, such as associative learning capacities (Cheng 2021, 2023; Bielecki et al. 2023; Botton-Amiot et al. 2023). Moon jellyfish, in particular, have emerged as a model organism in neurobiological research (Albert 2011; Pallasdies et al. 2019; Agrillo et al. 2025). However, to date, no study has specifically examined the aspects of social cognition involved in voluntary aggregation. Moon jellyfish were introduced into a novel tank with no shelter. At one end of the tank, two conspecifics were placed as social companions, while the opposite end remained empty. If the jellyfish tend to actively aggregate with conspecifics in this context, they are expected to swim toward and spend more time near the conspecifics.

## Methods

### Subjects

**Fig. 1:**
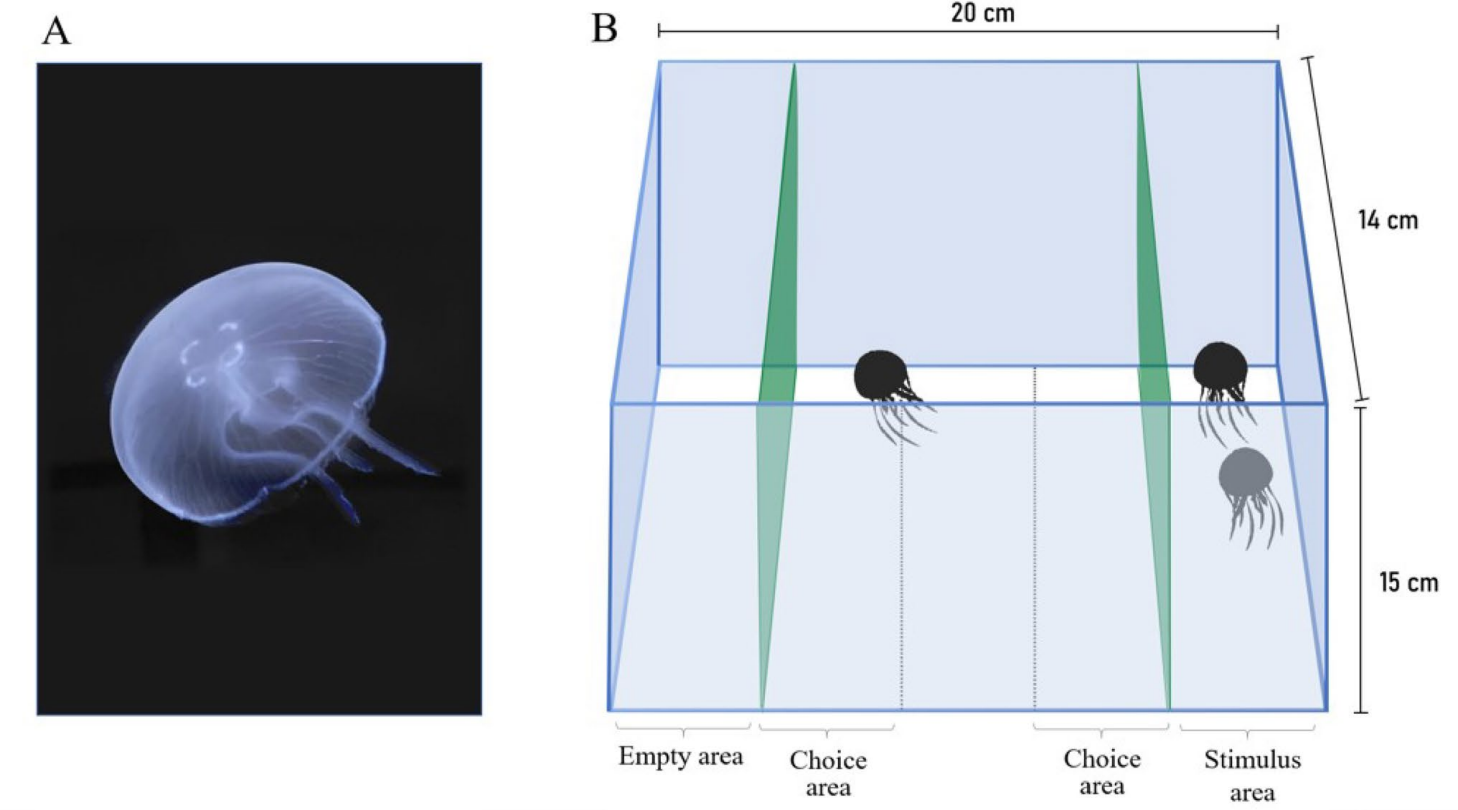
(**A**) One of the experimental subjects observed in this study (*Aurelia* spp). (**B**) Schematic representation of the experimental apparatus. The subject was singly inserted in the middle of the tank where two social companions were present on one side. As a measure of sociality, we recorded the time spent on the two opposite short sides of the tank (choice areas)

A total of 16 juvenile individuals of *Aurelia* spp. were tested (Fig. 1a). The jellyfish specimens used in this study were obtained from a Czech commercial supplier, with the animals originally collected from the Mediterranean Sea. The sex of the specimens was not determined, and no information was available regarding their original ecological context (e.g., shallow vs. deep-water habitat). Subjects were housed at the Department of General Psychology at the University of Padova in two identical stock tanks specifically designated for the maintenance of jellyfish (O16 Jellyfish aquarium). Each tank contained eight subjects. The tank is 38 cm tall, 20 cm deep, and includes 16.8 L. Salt water (salinity: 32 ppt; NO_3_ level: 20–25 mg/L) was maintained at a constant temperature of 22° ± 2 °C. The tank was circular in shape to prevent the jellyfish from getting tangled in the corners. Additionally, there was a constant clockwise water flow that facilitated the jellyfish’s swimming. The tank is equipped with built-in filtration and multi-LED lighting on the top (12 watts) and a remote control. Two hours before the experiment, moon jellyfish were fed to satiation with brine shrimps (*Artemia salina*). A 14-hour light/10-hour dark (L/D) photoperiod was used.

### Experimental apparatus and procedure

The experimental apparatus (length × height × width: 20 × 15 × 14 cm) was divided into five equal-sized sections along its length. The two outermost sections served as stimulus areas (4 × 14 cm), where social stimuli could be presented. Each stimulus area was separated from the adjacent inner sections by a green mesh (0.5 × 0.5 cm), which allowed the transfer of visual, chemical, auditory, and flow cues while preventing physical contact. The subject jellyfish was free to swim within the three inner sections. The central Sect. (4 × 14 cm) was defined as the neutral area, where the subject was placed at the beginning of each trial. The two sections adjacent to the stimulus areas were considered choice areas (4 × 14 cm). Time spent in a choice area was interpreted as an indication of preference for the corresponding stimulus.

The walls and floor of the tank were covered with white plastic material. The apparatus was illuminated by a single fluorescent lamp (6.5 watts), and the water level was maintained at 9 cm. Unlike the stock tanks, no pump was installed in the apparatus to prevent water flow from influencing the subject’s swimming direction.

Stimuli were introduced 5 min prior to the beginning of each test. The subject jellyfish was gently transferred using a jellyfish net into a transparent cylinder (6 cm in diameter) positioned in the neutral area of the tank. After a 2-minute acclimatization period, the cylinder was lifted, allowing the subject to swim freely. The jellyfish’s position within the three areas (the two choice areas and the central neutral area) was recorded over a 20-minute observation period.

Subjects were tested only once: In half of the trials (*N* = 8), the social stimuli were placed in the left stimulus area; in the other half (*N* = 8), they were placed in the right stimulus area to counterbalance potential side biases.

## Results

Statistical tests were carried out using SPSS 29.0.2.0. Data were normally distributed (Shapiro-Wilk, *p* = 0.240); accordingly, we used parametric analyses. First of all, we analyzed whether jellyfish preference changes over time and whether there was any side bias in the apparatus by a repeated-measures ANOVA with Time (five intervals of four minutes) as a within-subjects factor and Side of the stimuli (left/right) as a between-subjects factor. A one-sample *t*-test (0.50 as chance level) was used to assess the overall preference of jellyfish to swim in the two choice areas. This test was complemented with a Bayes Factor analysis to evaluate the strength of evidence supporting the alternative hypothesis over the null hypothesis (no preference for either stimulus area).

The repeated measures ANOVA showed that the proportion of time close to the social stimuli did not significantly change as a function of time (Time, *F*(4, 52) = 0.121, *p* = 0.975, *η*^*2*^_*p*_ = 0.009). The side of the stimuli (left vs. right) did not affect jellyfish choice (*F*(1, 13) = 2.536, *p* = 0.135, *η*^*2*^_*p*_ = 0.163). No interaction between Time and Side was found (*F*(4, 52) = 1.823, *p* = 0.138, *η*^*2*^_*p*_ = 0.123).

**Fig. 2:**
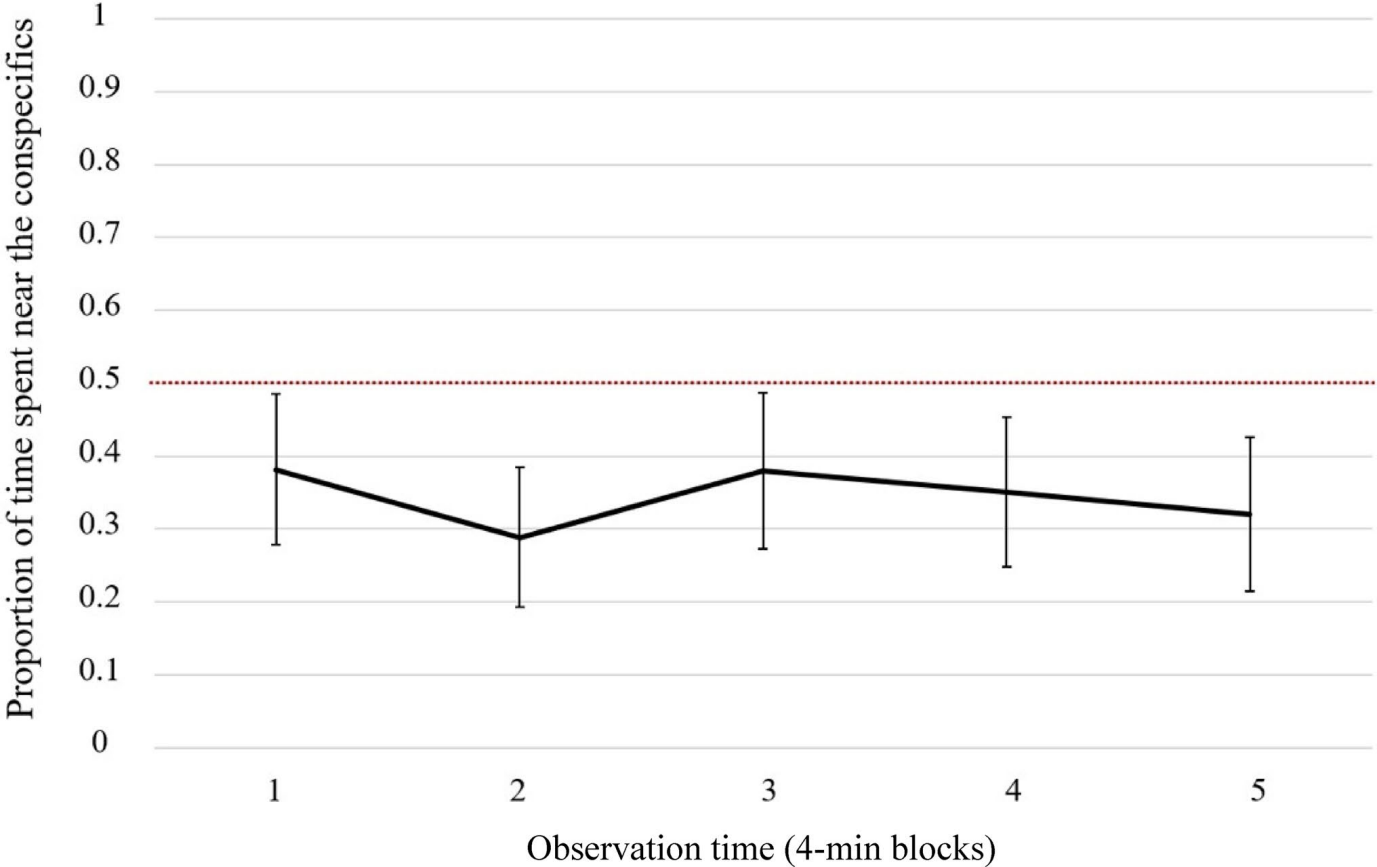
The proportion of time spent near the conspecifics was plotted against time (five intervals of four minutes). Moon jellyfish consistently spent more time in correspondence with the empty area. The dotted lines represent the chance level. Bars represent the standard errors of the mean

Overall, jellyfish did 13.81 ± 7.97 (mean ± std. dev.) movements across the three areas during the observation time and spent significantly more time near the empty compartment (*t*(15) = 2.541, *p* = 0.023; Cohen’s *d* = 0.635; Fig. 2; Table 1). The Bayes Factor indicated very strong evidence in favor of the alternative hypothesis (BF_10_ = 200; Jeffreys 1961).

**Table 1:** Time (sec) spent in the three central sections of the experimental tank

Subject	Time close to the conspecifics	Time in the central area	Time close to the empty sector
1	127	62	1011
2	972	138	90
3	86	207	907
4	79	148	973
5	510	265	425
6	342	144	714
7	480	156	564
8	544	401	255
9	359	286	555
10	269	767	164
11	298	267	635
12	296	276	628
13	336	249	615
14	34	141	1025
15	46	408	746
16	1	148	1051

## Discussion

We aimed to investigate whether jellyfish tend to associate with conspecifics when approaching a novel and potentially threatening environment, a hypothesis supported by substantial research on both vertebrates and invertebrates (Hager and Helfman 1991a, b; Nieder 2020). Both inferential and Bayesian analyses provide clear evidence that jellyfish can perceive the presence of conspecifics and adjust their movements accordingly. However, contrary to our initial hypothesis, jellyfish did not preferentially swim toward the area containing their conspecifics. Instead, they spent significantly more time near the empty compartment. This preference remained consistent throughout the observation period and was not influenced by any side bias in the apparatus.

The non-social behavior exhibited by moon jellyfish in this context suggests that the jellyfish aggregation observed in the wild may result more from environmental factors (e.g., sun-based navigation, food resource distribution) than from an active drive to associate with conspecifics. Moreover, this behavior suggests that the anti-predator strategy employed by moon jellyfish — namely, the use of venom delivered by nematocysts — may be highly effective and energetically efficient. This strategy might reduce the evolutionary pressure to adopt additional group-based defense mechanisms, such as the ‘safety in numbers’ effect.

It is well established that animals’ social behavior is strongly influenced by environmental conditions (reviewed in Krause and Ruxton 2002). For instance, animals raised with limited sensory stimulation (such as stimulus deprivation and poor environmental enrichment) often exhibit reduced environmental exploration and tend to withdraw from social interactions (Sackett 1965). Since there is no specific literature on sensory enrichment for jellyfish, we can only speculate on its potential effects. In contrast to other species typically examined in animal welfare research, jellyfish lack a centralized brain, making it challenging to define appropriate laboratory conditions or meaningful forms of environmental enrichment.

Nonetheless, our subjects were housed in groups of eight individuals per tank, ensuring social exposure before testing. Furthermore, the jellyfish displayed active exploration of the environment, making an average of 14 movements across the three areas of the apparatus, despite the absence of any water flow. Lastly, we emphasize that jellyfish were not food-deprived before testing. Although the tendency to avoid conspecifics when searching for food has been reported in other species (Dill and Fraser 1984; Hoare et al. 2000), this explanation is unlikely to account for the observed preference for the empty compartment.

It is worth noting that our experimental apparatus differed from those commonly used in fish studies. In such studies, the stimulus area is often located in a separate but adjacent tank, allowing only visual cues to reach the test subjects. Moon jellyfish are known to respond to light (Hamner et al. 1994; Arai 1997), but their visual system is primitive. For this reason, we set up an apparatus that allowed both visual and chemical communication between the stimulus and the subject jellyfish. We cannot exclude the possibility that the stimulus jellyfish released alarm-related chemical cues, leading the test subject to avoid that area. For instance, if moon jellyfish are not highly social in such contexts, the stimuli individuals may have experienced stress due to the enforced proximity of conspecifics, possibly triggering the release of aversive chemical signals. Additionally, we were unable to determine the sex of the subjects and the stimuli. Therefore, we cannot exclude the possibility that the non-social behavior observed in our subjects was influenced by an uneven distribution of sexes between the stimuli and the subject jellyfish. Male stimuli, for instance, may have released sex-specific chemical cues that repelled other males.

The embodied cognition framework proposes that cognitive processes are grounded in the sensorimotor structures of the body and interactions with the environment. Goldman and De Vignemont (2009) argue that social cognition depends on ‘bodily formats’, meaning representations that are directly tied to perceptual and motor systems, rather than being purely abstract or amodal. Wehrle (2020) further distinguishes between the lived experience of embodiment (being a body) and the physical body as an object (having a body), emphasizing the temporal and situated nature of embodied intentionality. Given that jellyfish lack a central brain, we believe that our results may pave the way to a deeper understanding of the evolutionary foundations of embodied intentionality and basic forms of social cognition.

In conclusion, we recognize that a single experiment is insufficient to settle a complex issue such as whether a species tends to approach or avoid conspecifics. Preliminary investigations are often more valuable for highlighting new questions than for providing definitive answers. We did not identify the sensory mechanisms by which jellyfish detect conspecifics in novel environments, and even under the assumption of chemical communication, the specific messengers used in our experimental context remain unknown. In addition, the lack of precise information about the collection site of the studied population limits the generalization of our findings to other ecological contexts. Despite these limitations, our results provide clear evidence that moon jellyfish detect conspecifics and actively move away from them in unfamiliar environments, a behavior that may involve a simple decision-making process within their nervous system.

We suggest that the aggregations of moon jellyfish observed in natural habitats may result more from ecological factors, such as water currents, rather than from coordinated and active social behavior. Future studies are needed to elucidate the mechanisms underlying the perception of conspecifics in jellyfish and to explore the potential reasons for avoiding social proximity under certain conditions.

## Supplementary Information

Below is the link to the electronic supplementary material.

Supplementary Material 1

## Data Availability

Data are available in the supplementary material.
